# ADCC-mediating non-neutralizing antibodies can exert immune pressure in early HIV-1 infection

**DOI:** 10.1371/journal.ppat.1010046

**Published:** 2021-11-17

**Authors:** Dieter Mielke, Gama Bandawe, Jie Zheng, Jennifer Jones, Melissa-Rose Abrahams, Valerie Bekker, Christina Ochsenbauer, Nigel Garrett, Salim Abdool Karim, Penny L. Moore, Lynn Morris, David Montefiori, Colin Anthony, Guido Ferrari, Carolyn Williamson

**Affiliations:** 1 Department of Surgery, Duke University, Durham, North Carolina, United States of America; 2 Institute of Infectious Diseases and Molecular Medicine and Division of Medical Virology, University of Cape Town, Cape Town, South Africa; 3 Malawi University of Science and Technology, Thyolo, Malawi; 4 University of Alabama at Birmingham, Department of Medicine, Birmingham, Alabama, United States of America; 5 National Institute for Communicable Diseases, Johannesburg, South Africa; 6 Centre for the AIDS Programme of Research in South Africa (CAPRISA), University of KwaZulu Natal, Durban, South Africa; 7 Discipline of Public Health Medicine, School of Nursing and Public Health, University of KwaZulu Natal, Durban, South Africa; 8 Department of Epidemiology, Columbia University, New York, New York, United States of America; 9 University of Witswaterstrand, Johannesburg, South Africa; 10 National Health Laboratory Service, Johannesburg, South Africa; Emory University, UNITED STATES

## Abstract

Despite antibody-dependent cellular cytotoxicity (ADCC) responses being implicated in protection from HIV-1 infection, there is limited evidence that they control virus replication. The high mutability of HIV-1 enables the virus to rapidly adapt, and thus evidence of viral escape is a very sensitive approach to demonstrate the importance of this response. To enable us to deconvolute ADCC escape from neutralizing antibody (nAb) escape, we identified individuals soon after infection with detectable ADCC responses, but no nAb responses. We evaluated the kinetics of ADCC and nAb responses, and viral escape, in five recently HIV-1-infected individuals. In one individual we detected viruses that escaped from ADCC responses but were sensitive to nAbs. In the remaining four participants, we did not find evidence of viral evolution exclusively associated with ADCC-mediating non-neutralizing Abs (nnAbs). However, in all individuals escape from nAbs was rapid, occurred at very low titers, and in three of five cases we found evidence of viral escape before detectable nAb responses. These data show that ADCC-mediating nnAbs can drive immune escape in early infection, but that nAbs were far more effective. This suggests that if ADCC responses have a protective role, their impact is limited after systemic virus dissemination.

## Author summary

Reponses that clear or control viral replication in acute infection are considered important responses to elicit by vaccination. In the only HIV-1 vaccine trial to date to show any efficacy, antibody-dependent cellular cytotoxicity (ADCC)-mediating non-neutralizing antibodies (nnAbs) were correlated with reduced risk of infection. However, the role of ADCC-mediating antibodies in controlling replication in acute infection is not well understood. Viral escape provides evidence of the importance of immune responses, but there is very little evidence of HIV-1 immune escape from nnAbs to-date. We assessed the impact of early ADCC-mediating nnAbs on HIV-1 *envelope* evolution in infected individuals where we had early samples, prior to the detection of nAbs. We found evidence of escape from ADCC-mediating nnAbs, but in only one individual. In contrast, escape to nAbs was rapid and with or without resistance to ADCC-mediating antibodies. We identified escape pathways which were sensitive to neutralization but resistant to ADCC. These data show that ADCC-mediating nnAbs can drive immune escape in early infection, but to a lesser extent than nAbs, suggesting they may have a limited protective role after systemic virus dissemination.

## Introduction

Non-neutralizing antibodies (nnAbs) have been correlated with protection for many licenced vaccines [1]. In the RV144 vaccine trial, protection against HIV-1 infection was associated with Envelope (Env) variable loop V2-directed nnAbs [2, 3]. Secondary correlates analysis of this trial found that high levels of antibody-dependent cellular cytotoxicity (ADCC) activity, together with low levels of anti-Envelope (Env) IgA responses, inversely correlated with risk of infection. A sieve effect which may have been due to ADCC-mediating nnAbs provided further evidence of their importance [4].

In addition to their potential role in protection, numerous studies provide substantial evidence that ADCC-mediating antibodies play an important role in the context of HIV and SIV infection. Non-human primate and human studies have associated ADCC responses with lower viral loads [5–13], protection from infection [14, 15], improved clinical outcome in linked transmission [12], and reduced mother-to-child transmission [16]. Moreover, we recently identified an instance where ADCC-mediating nnAbs were able to limit nAb escape in an HIV-infected individual [17].

However, there is limited direct evidence for ADCC playing a role in controlling viral replication in primary HIV-1 infection [18, 19]. Only one study to date, of individuals with chronic infection, has clearly demonstrated HIV-1 escape from ADCC [20]. Confirmation of viral escape from ADCC-mediating nnAbs in humans following infection, such as has been demonstrated in mouse models [21], would be an important indicator of their importance as an immune response.

HIV-specific cytotoxic T-lymphocyte (CTL) responses are detected early in acute viraemia, and are associated with viraemic control [22]. NnAb responses are detected soon after CTLs [23], and usually emerge earlier than neutralizing antibody (nAb) responses which evolve several weeks to months after infection [17]. Following infection, both CTLs and nAbs exert strong immune pressure on the virus, forcing rapid escape [22, 24, 25]; however there is no evidence that ADCC has a similar impact. A major challenge in characterising ADCC-mediating nnAb immune pressure is that it is difficult to deconvolute Fc-mediated ADCC function from Fab-dependent neutralization function once nAbs have developed. Here, in women recruited very early in HIV-1 infection with detectable ADCC responses prior to the development of nAbs, we investigated whether ADCC-mediating nnAbs can impact viral populations.

## Results

### ADCC-mediating nnAb responses can exert immune pressure in early infection

**Fig 1:**
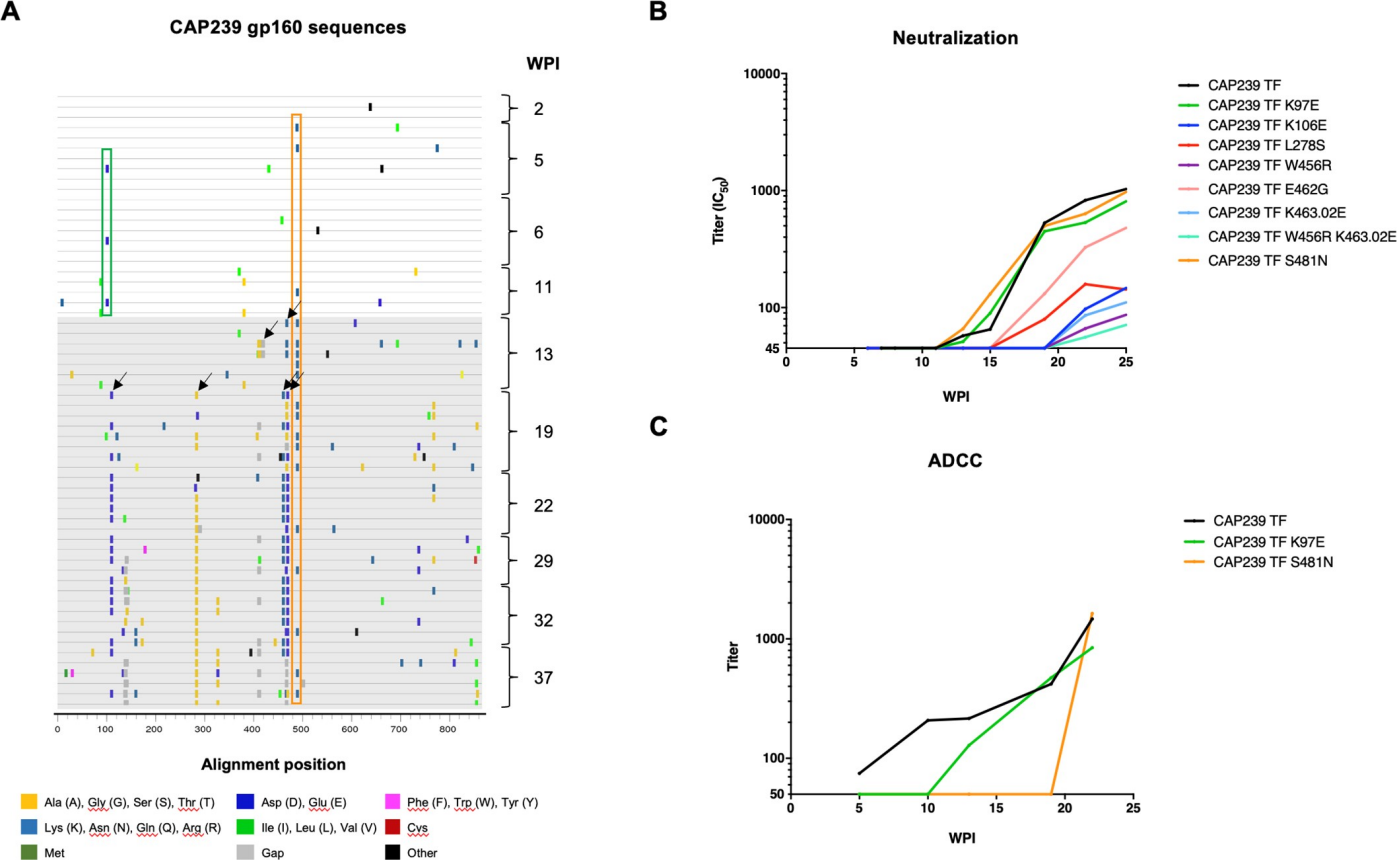
Escape from ADCC and neutralizing antibody responses in CAP239. **(A)** Highlighter amino acid plot comparing 61 single genome *env* sequences, generated from 10 time-points over eight months of infection, to the T/F sequence (top line). The two sites that were evolved prior to nAb responses, and occurred in multiple sequences, are shown as boxes: K97E (green box), and S481N (orange box); and the eight sites/combination sites that evolved after nAb responses, and occurred in multiple sequences, are as arrows (K97E, K106E, L278S, W456R, E462G, K463.02E, S481N, W456R K463.02E). **(B)** Neutralization titers (IC_50_) of longitudinal autologous plasma evaluated against pseudoviruses: CAP239 T/F *env*, and 10 CAP239 T/F with sites/combination sites which evolved after nAb responses developed. **(C)** ADCC antibody titers against infected targets cells in the Luciferase ADCC assay. Cells were infected with T/F virus, as well as the T/F virus with K97E or S481N incorporated. These viruses were used to infect CEM.NKR_CCR5_ as targets and utilized with donor PBMCs as effectors. WPI indicates weeks post infection. Points on ADCC and neutralisation plots indicate the mean of ≥three independent experiments.

In a cohort of recently infected women from the CAPRISA 002 acute infection cohort, we were interested in identifying the role of ADCC in driving early escape in the gp160, prior to the detection of nAbs. One participant, CAP239, was recruited within two weeks of HIV-1 infection (HIV-1 antibody negative/RNA positive). This individual was infected with a single transmitted/founder (T/F) virus, with CTL responses mapped to Gag, Pol, Vpr, Nef and the Env gp41 [22]. ADCC responses to the T/F virus were detected in the participant’s plasma at five weeks post-infection (wpi), while neutralizing antibody responses were detected at 13 wpi (Table 1). To identify sites under antibody pressure, we compared *env* sequences from ten time-points (2–37 wpi). Prior to the detection of nAb responses, two sites were identified in samples from 5–11 wpi as putative ADCC escape mutations, distinct from previously mapped CTL responses [22]: K97E (all amino acid positions are labelled using the reference strain HxB2) which was present in 3/19 sequences; and S481N which was present in 3/19 sequences (Fig 1A). After nAbs were detected (13 wpi), S481N increased in frequency (16/39 sequences from 13–37 wpi), and two additional sites appeared under immune pressure (a three-residue deletion at positions 405 to 407, and position 462). From 19 wpi, amino acid toggling was observed at four additional sites (106, 278, 456 and 463.02).

**Table 1:** Characterisation of autologous ADCC and nAb responses in five CAPRISA 002 participants.

Participant	ADCC responses present (wpi)	nAbs first detected (wpi)	Target of first nAbs
**CAP239**	≤ 5	13	CD4bs
**CAP45**	≤ 5	9	V5
**CAP210**	≤ 5	16	V1V2
**CAP63**	≤ 4	7	V4
**CAP88**	≤ 5	11	C3

ADCC assays required generating infectious molecular clone (IMC) mutants. Making IMCs is time consuming and we thus first confirmed mutations associated with nAb escape by mapping nAb epitopes. Sites of interest, that were not associated with neutralization escape, were then investigated as potential ADCC escape epitopes.

First, to map escape from nAbs, we introduced the observed mutations into the CAP239 T/F *env* and tested the effect of each mutation/combination on neutralization by autologous plasma using the TZM-bl pseudovirus (PSV) neutralization assay (Fig 1B). Of the seven single/one double mutants we tested, five became resistant to autologous nAbs, all of which were located in the CD4 binding site (CD4bs) or CD4bs-targeting bnAb contact sites (K106E, W456R, E462G, K463.02E and W456R K463.02E). Importantly, however, the two early (5–11 wpi) mutants K97E (a VRC01 contact site in the C1) and S481N (a residue of the α5-helix of layer 3 in the C5) had no effect on neutralization susceptibility.

To investigate escape from ADCC-mediating antibodies, these two early mutations (K97E and S481N) were introduced into the CAP239 T/F Env-IMC-LucR reporter virus. The K97E mutation (CAP239 T/F K97E) resulted in complete resistance to ADCC mediated by autologous plasma until 10 wpi, after which ADCC responses to the mutant were detected (Fig 1C). Similarly, the S481N mutation (CAP T/F S481N) was associated with escape from ADCC until 22 wpi, at which time it became sensitive to plasma antibody-mediated ADCC responses. Significantly, these mutations showed similar ADCC susceptibility to purified IgG from plasma of chronically infected individuals (HIVIG), and binding of HIVIG antibodies to infected cells, CD4 downregulation and infectivity (S1 Fig), indicating cells infected with these viruses had similar Env expression on the cell surface and the two mutations did not substantially affect Env conformation. Thus, we conclude that ADCC responses were responsible for driving escape at these two sites.

### ADCC-mediating nnAb and nAb responses drive viral evolution along distinct pathways

**Fig 2:**
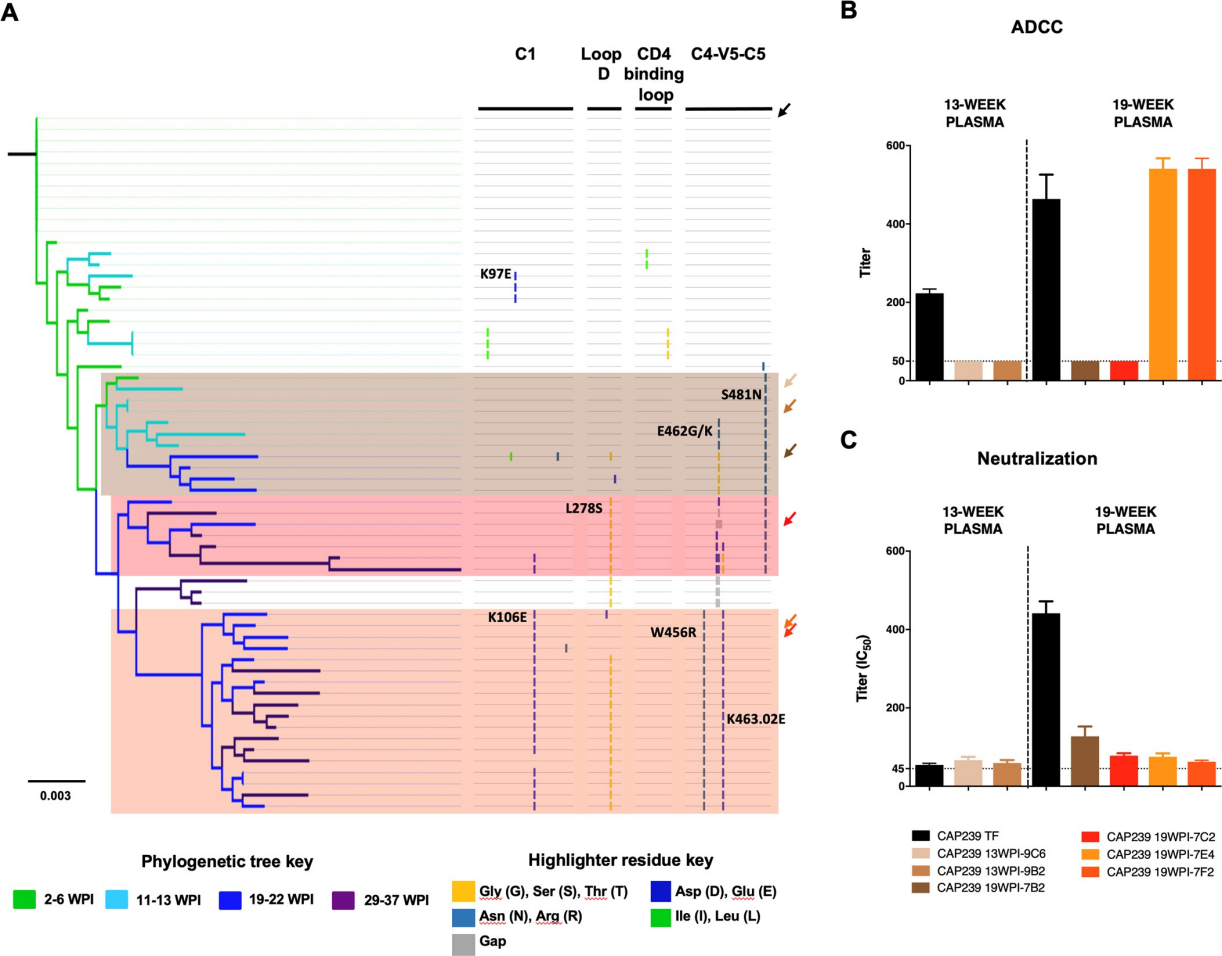
Impact of ADCC and neutralization responses on evolutionary pathways in CD4-binding site regions of CAP239 viruses. **(A)** A maximum-likelihood tree of the 61 CAP239 full-length *env* single genome sequences was constructed, and rooted on the T/F virus. Branches were colour-coded according to the time-point from which the sequence was sampled. Three distinct evolutionary pathways were observed following detection of nAbs. Arrows indicated sequence used to generate infectious molecular clones containing the *env* of the T/F (black) as well as six representative sequences from the three pathways were constructed (identified by arrows coloured according to pathway). Two of the clones (CAP239 13WPI-9C6 and CAP239 13WPI-9B2) were representative of 13 week viruses; while four viruses (CAP239 19WPI-7B2, CAP239 19WPI-7C2, CAP239 19WPI-7F2 and CAP239 19WPI-7E4) represented 19 week viruses. **(B)** Env-IMCs were tested against contemporaneous plasma was tested for ADCC activity. **(C)** Env-IMCs were tested against contemporaneous plasma for neutralization activity. Points on ADCC and neutralisation plots indicate the mean of ≥three independent experiments.

It was evident that while both ADCC and nAb responses targeted regions in or proximal to the CD4 binding site, the epitopes were different. We were thus interested whether ADCC and nAb responses differentially impacted *env* evolution over the first six months of infection. A maximum-likelihood phylogenetic tree of CAP239 full-length *env* sequences revealed three distinct pathways that emerged before, or at the time of, nAb detection (13 wpi) (Fig 2A). Viruses incorporating K97E were found in a dead-end pathway that did not persist beyond 6 wpi, so we did not explore this mutation further. Instead, we incorporated the T/F *env* (black) and six *envs* representative of the three major evolutionary pathways into Env-IMC-LucR (Fig 2), and screened these for sensitivity to ADCC and nAb responses.

Pathway one (brown) contained viruses from 5, 13 and 19 wpi, and was characterized by escape in the C4-V5-C5 region. Viruses from this pathway containing the S481N mutation (detected at 13 wpi) were resistant to ADCC mediated by contemporaneous plasma but sensitive to neutralization by nAb responses. However, while viruses containing both E462K/G and S481N (present at 19 wpi) remained resistant to contemporaneous ADCC responses, they were also partially resistant to nAb responses compared to the CAP239 T/F at 19 wpi. Pathway one was a dead-end evolutionary pathway as it was not detected after 22 weeks.

The second (red) and third (orange) pathways contained viruses from later infection (19 to 37 weeks). Pathway two was characterized by mutations in loop D (L289S), the C5 (S481N), as well as deletions and toggling in the V5 (at positions 461–463.02), while pathway three (orange) was characterized by mutations in the C1 (K106E), loop D (L278S) and C4-V5 (W456R and K463.02E) regions. While the Env-IMC-LucR virus encoding an *env* from pathway two was resistant to both ADCC and nAb responses present at 19 wpi, the viruses encoding *env* from pathway three were sensitive to ADCC by contemporaneous plasma but were resistant to nAbs (Fig 2B and 2C). Together these results demonstrate that ADCC responses are moulding viral populations in early infection, however this is not maintained in later infection when nAb escape pathways dominated.

**Fig 3:**
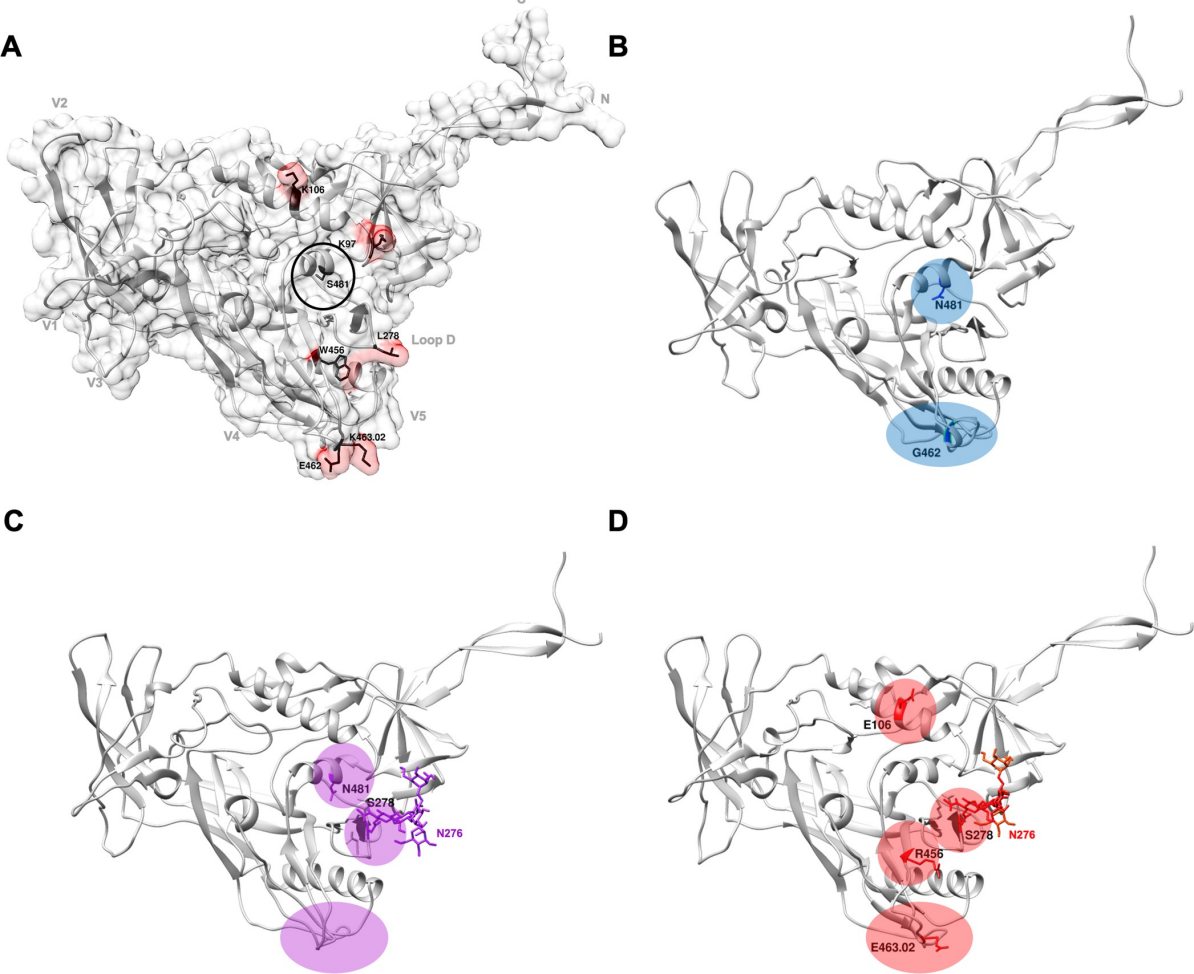
Models of the CAP239 gp120: effect of ADCC and neutralization escape mutations on Env structure. Trimer models of CAP239 T/F and Envs from each of the three evolutionary pathways were constructed using Modeller [53] and the best scoring model was visualised using Chimera [54]. A single gp120 monomer from the trimer of the T/F or Envs from each pathway were used for further analysis. GlyProt [55] was used to add basic glycans (N276 in pathway two and three). **(A)** The contact surface was modelled onto the CAP239 T/F gp120 (grey) with any key residue surfaces shown in red. Structural landmarks (grey) and key residues in CAP239 viral evolution (black) are labelled on the CAP239 TF gp120. Changes characteristic of evolution are shown in **(B)** blue for pathway one (CAP239 19-7F4), **(C)** purple for pathway two (CAP239 19-7C2), and **(D)** red for pathway three (CAP239 19-7H2; pathway three).

To further characterise ADCC and nAb epitopes, the CAP239 T/F Env trimer, as well as Envs characteristic of each escape pathway, were modelled onto the most representative trimer structure available and a single gp120 monomer was then used for analysis (Fig 3). All pathways exhibited structural or charge changes in the V5: in pathway one, E462G resulted in a small helix in the V5; in pathway two, the V4 and V5 became less structured (including the loss of a β sheet in the V4), possibly due to a three-residue deletion in the V5; pathway three introduced a charge reversal in the V5 (K463.02E). In addition, a glycan was added at N276 in pathways two and three, which may have shielded the antibody epitope. Pathway three also introduced W456R in the β23 sheet, and a charge change (K106E) in the C1. Of interest, S481N (α5-helix of layer 3), which escaped ADCC responses and was observed in pathways one and two (resistant to ADCC), was not accessible to antibodies targeting the Env trimer (Fig 3A, circled in black).

### Limited changes in Env after ADCC-mediating nnAb, and prior to nAb, responses

To determine if we could find evidence of ADCC pressure in other acutely infected individuals, we evaluated viral escape in four additional CAPRISA 002 women (CAP45, 63, 88 and 210). These participants were recruited within two to five weeks post infection (wpi). All participants were infected with a single T/F virus, had detectable ADCC responses directed against the T/F Env at enrolment, and developed nAb responses seven to 16 weeks after enrolment. The target of the first nAb responses varied between participants, and was restricted to one region of the Env (Table 1) (V5, V1V2 V4 and C3 for CAP45, 210 63 and 88, respectively), as we previously reported [25].

**Fig 4:**
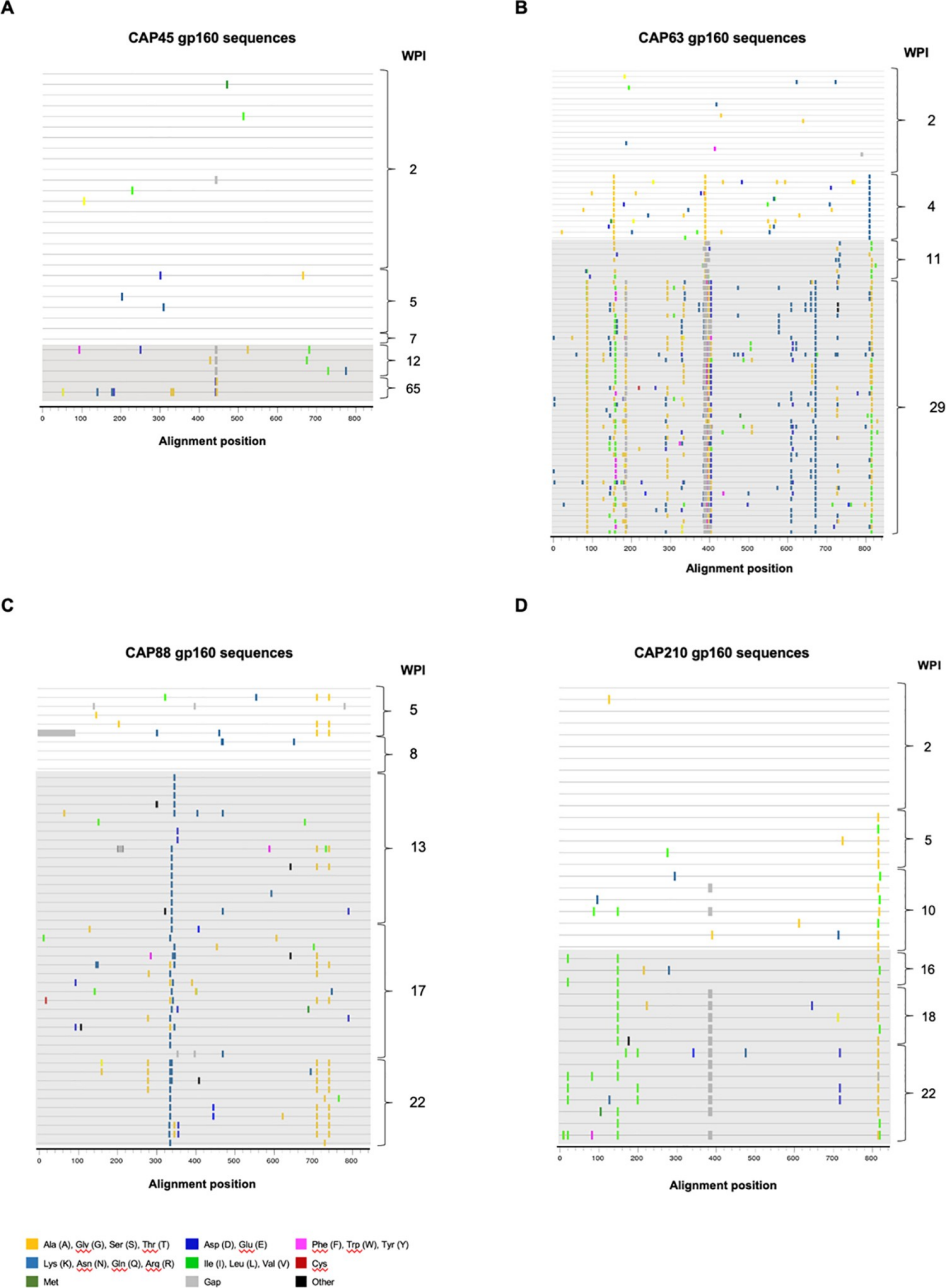
Highlighter amino acid plots showing viral evolution of the HIV-1 *envelope* in five CAPRISA 002 participants. Full-length *env* sequences were generated over time for five CAPRISA 002 participants. In CAP45, 31 single genome sequences were obtained from five time-points over 1 year of infection **(A)**; in CAP63, 84 single genome sequences were obtained from four time-points over six months of infection **(B)**; in CAP88, 52 single genome sequences were obtained from five time-points over six months of infection **(C)**; and lastly, in CAP210, 39 single genome sequences were obtained from six time-points over six months of infection **(D)**. The presence of detectable nAbs is indicated by grey shading. The reference sequence for each participant is the respective T/F sequence.

Here, we investigated sites under putative immune pressure over the entire *env* (Fig 4), using 209 *env* sequences generated from infection to after the development of nAb responses (an average of 52 per participant, range: 31–82). We identified limited changes in Envelope prior to nAb responses: in two individuals there were either no sites under selection (CAP45), or where selection was observed this could be accounted for by proven CTL responses (CAP210) (A825V/T and T826A) [22]. Low frequency, conserved changes were identified in the remaining two participants (CAP63 and CAP88). In CAP63, escape was observed in V2 (A151T), V4 (P397S) and gp41 (D818N) and in CAP88, there were two sites under selection in the cytoplasmic tail which were present at the first time-point (G723S and S754A).

### Viral escape results in resistance to both neutralizing and ADCC-mediating antibodies

We constructed infectious molecular clones (Env-IMC-LucR) encoding the *env* of the T/F viruses and putative escape viruses and evaluated the effect of the identified mutations on both nAb and ADCC sensitivity. Of the four participants, two were evaluated in detail: CAP45 and CAP210. CAP63 was excluded as we have previously shown that changes in the V4 loop resulted in escape from both nAb and ADCC responses [17], while CAP88 was excluded as the changes of interest (in the cytoplasmic tail) could not be incorporated into the construct used for this ADCC assay.

**Fig 5:**
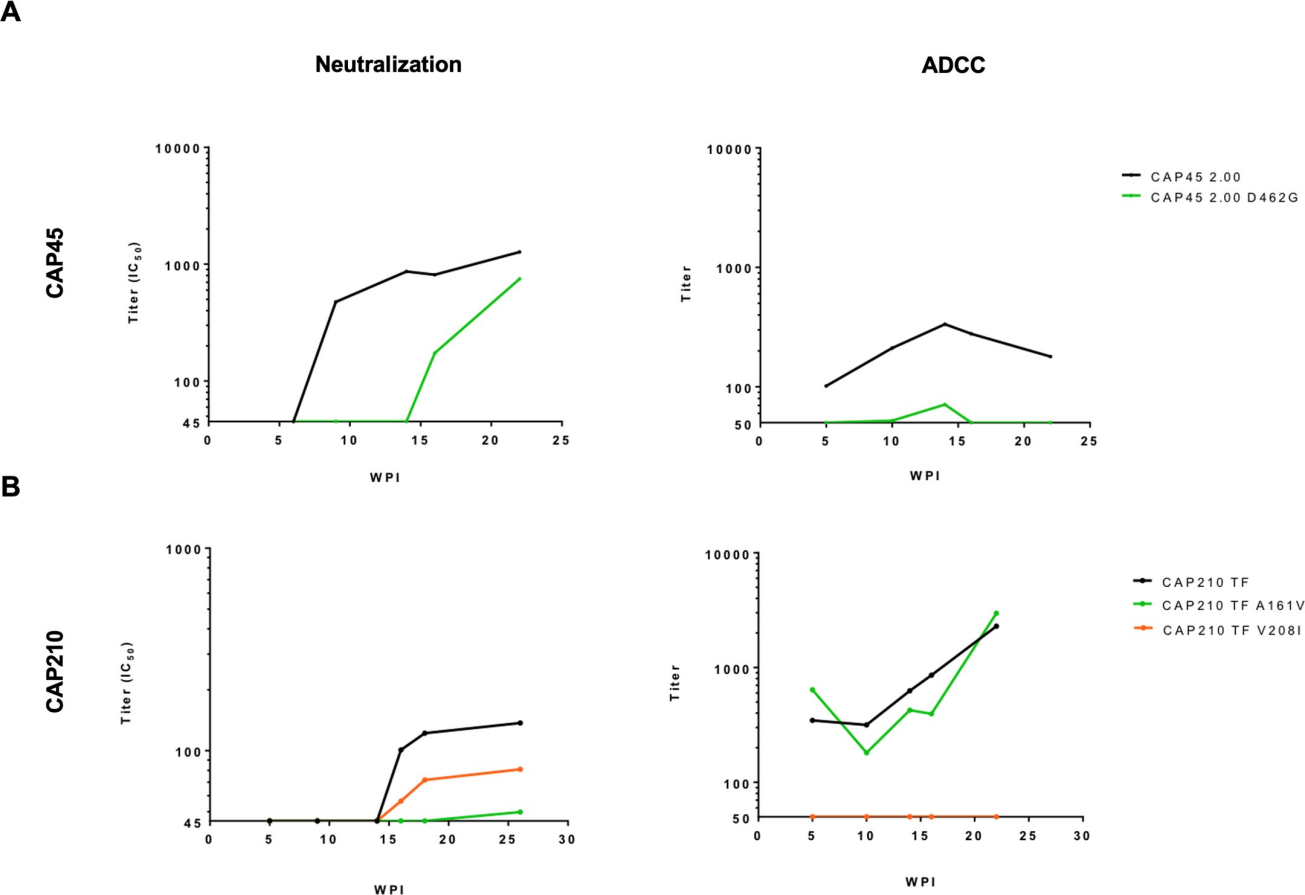
Escape from both neutralization and ADCC responses in two CAPRISA 002 participants. Neutralizing and ADCC responses were evaluated against wildtype and mutant viruses where putative ADCC or nAbs escape mutations were introduced into the autologous CAP45 or CAP210 T/F Env-IMCs. The impact of each mutation on sensitivity to autologous nAb and ADCC responses was then tested using longitudinal plasma. **(A)** Effect of the D462G mutation on neutralization and ADCC responses in CAP45; **(B)** Effect was A161V and V208I mutations on nAb and ADCC responses. Points on ADCC and neutralisation plots indicate the mean of ≥three independent experiments.

In CAP45, *env* sequences from 2–7 wpi were highly homogenous, with stochastic mutations consistent with Reverse Transcriptase error. However, at 12 wpi after the detection of nAbs, a three-residue deletion in the V5 (HxB2 positions 460–462, 460Δ3) was identified, and by 65 wpi the viruses harbouring the deletion was not detected in the sequences generated, having been replaced by viruses with two mutations in V5: K460E and D462G (Fig 4A). We further performed quantitative deep sequencing of the region spanning the nAb epitope, using the Primer ID method [26], to investigate the kinetics of escape further. This revealed the existence of this virus with the 460Δ3 deletion (10% of the viral population) at 9 wpi, the time of nAb detection, and increased to 23% at 10 wpi and 60% by 12 wpi, after which it decreased to 45% at 14 wpi. In addition, D462G was detected at very low levels (1.5%) at 10 wpi and increased to 11% at 12 wpi and 49% at 14 wpi (S2 Fig). To determine the impact of these mutations on ADCC and nAb sensitivity, the three-residue deletion and D462G were incorporated into the acute CAP45 Env-IMC-LucR backbone (CAP45 2.00). While the Env-IMC-LucR virus containing 460Δ3 was not able to infect target cells at levels sufficient for the ADCC assay, the D462G mutant Env virus (CAP45 2.00 D462G), induced resistance to both ADCC and nAb (until 15 wpi) responses (Fig 5A).

In the second participant, CAP210, we identified four regions under putative pressure, namely A161V in V2, V208I in C2, a six-residue deletion in V4, and several sites in the cytoplasmic domain of gp41 (Fig 4D). Deep sequencing of the C1-C2 regions revealed that both A161V and V208I were detected several weeks before nAbs emerged (A161V was present in 10% of variants at 12 wpi, while V208I was present in 15% of variants at 12 wpi) (S2 Fig). A161V became dominant, concurrent with the detection of nAbs, being present in ~ 80% of the viral population, while V208I remained a minor variant (present in 10–20% of the viral population from 16–26 wpi). Both mutations were incorporated into the CAP210 T/F Env-IMC-LucR backbone, and their sensitivity to ADCC and nAbs was investigated. CAP210 T/F A161V mutation did not have any effect on ADCC sensitivity, but conferred resistance to neutralization (Fig 5B). In comparison, CAP210 T/F V208I was completely resistant to plasma-mediated ADCC responses and was partially resistant to nAbs.

### Neutralization escape at very low nAb titers

**Fig 6:**
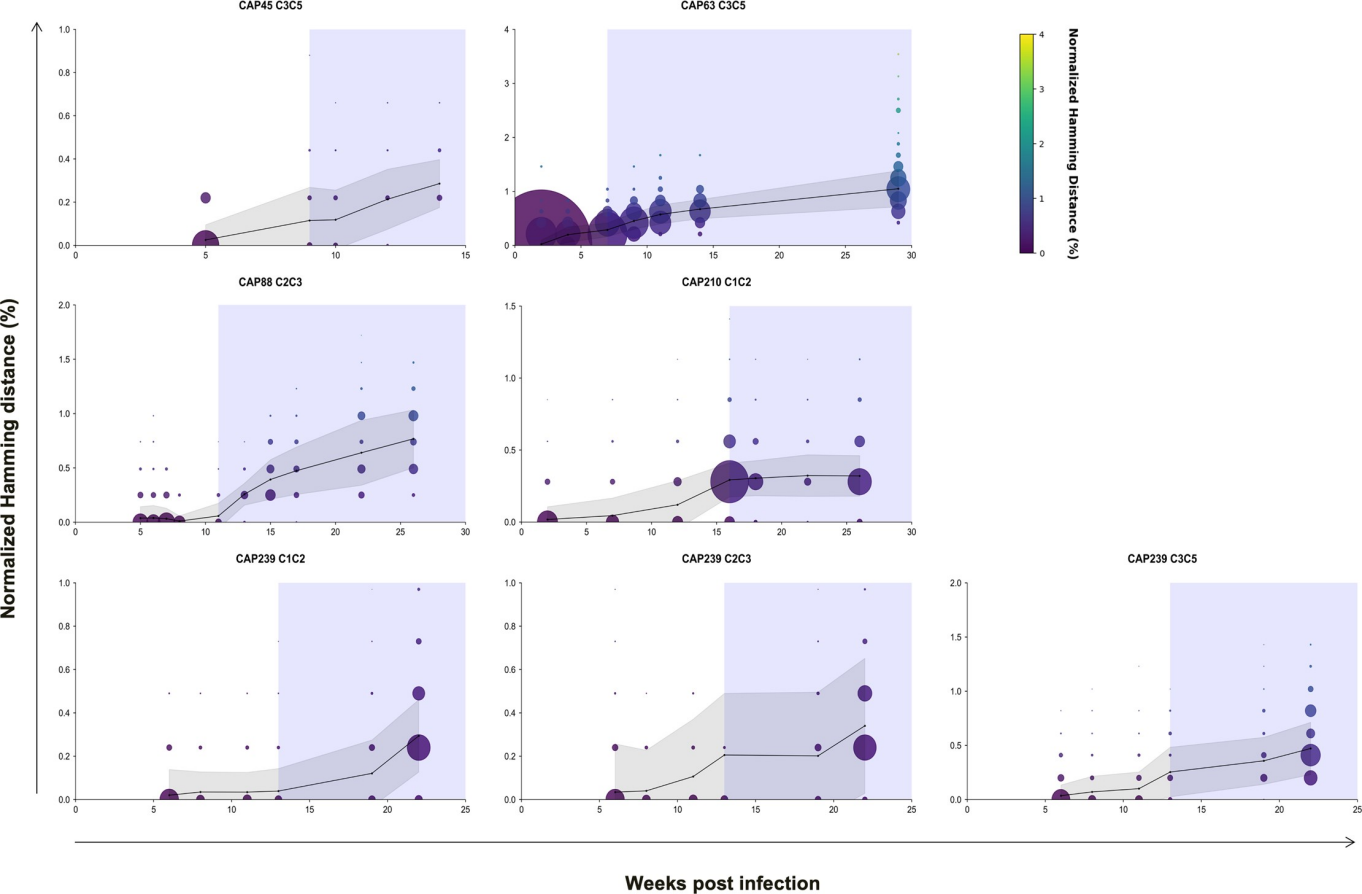
Divergence from T/F sequences in regions targeted by the initial autologous nAb responses in five CAPRISA 002 participants. A total of 51–12842 consensus sequences were generated per time point (an average of 1493 consensus sequences per time point) using Illumina deep sequencing platform and the primer ID method. Average hamming distances, normalised to the nucleotide length of each amplicon, were calculated for sequences generated at each time-point (black line). The bubble size indicates the size of the viral population, scaled by viral load. The shaded region indicates the time from which the initial autologous antibody response was first detected (IC_50_). Hamming distances were adjusted that so deletions were calculated as one event.

**Fig 7:**
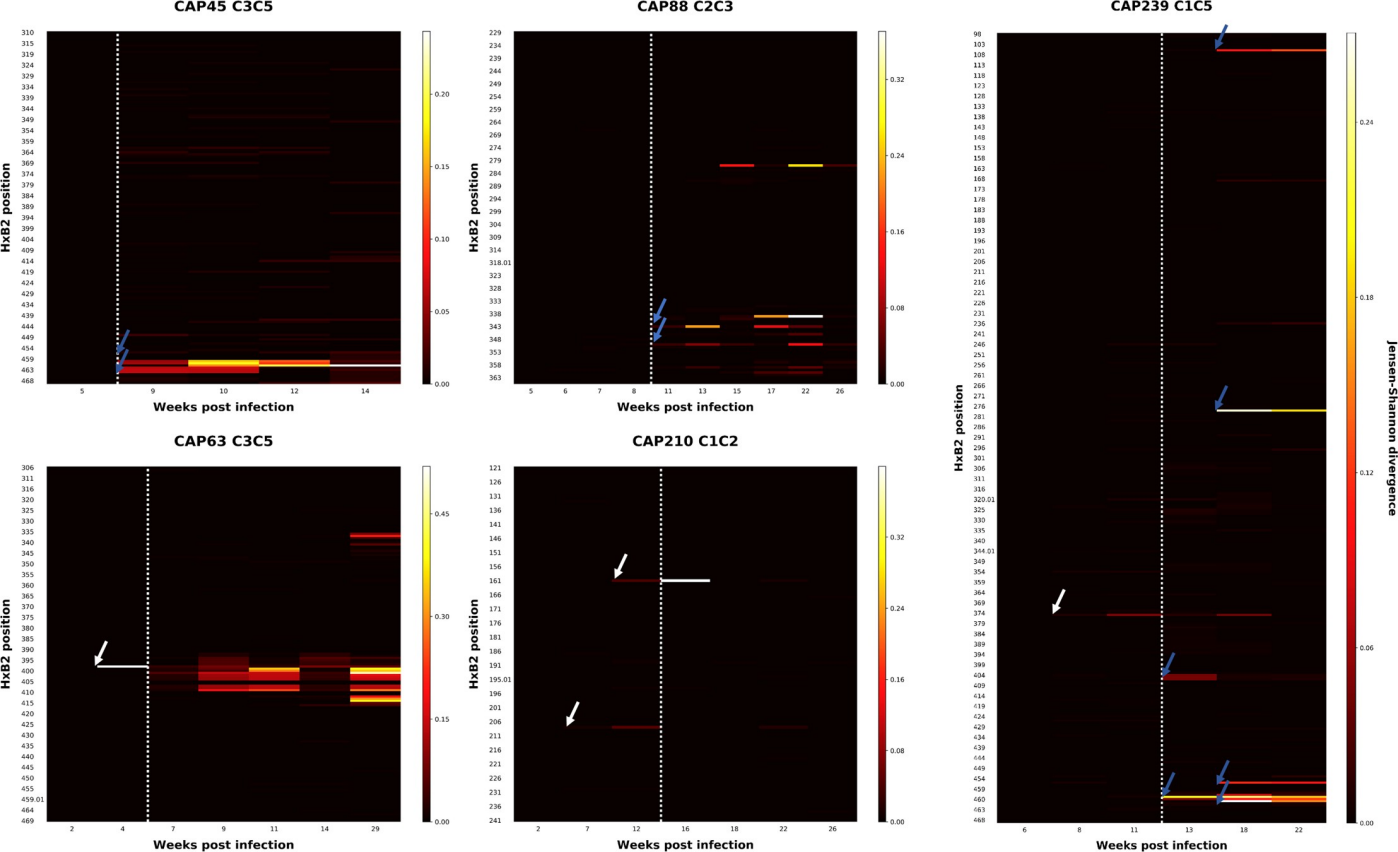
Jensen-Shannon Divergence (D_JS_) plots of the region targeted by initial autologous nAb responses in five CAPRISA 002 participants. D_JS_ for each position at each time-point sequenced was calculated. White arrows indicate sites exhibiting high D_JS_ prior to detectable nAbs: one site (position 397) in CAP63, two sites (161 and 208) in CAP210, and one site in CAP239 (365). Sites exhibiting raised D_JS_ after the detection of nAbs are identified using blue arrows: three sites (positions 460–462) in CAP45, two sites (positions 343 and 350) in CAP88 and five sites (106, 278, 459, 463 and 463.02) in CAP239, which were identified as a second wave of sites which diverged later (blue arrows). The white dotted line indicates the time from which the initial autologous antibody response was first detected (Titers >1:50).

Our study found that ADCC driven escape is rare, and the rapid escape to nAbs confirms that these antibodies exert more pressure on the virus. Using single genome and deep sequencing, we tracked the impact of early ADCC and nAb responses on viral *env* evolution. We determined the rate of *env* divergence from the T/F sequence (from enrolment) to the last nAb negative time-point (% normalised Hamming distance per month), and then from the last nAb negative time-point to the time-point of first detectable nAbs. In four of the five individuals, there was low frequency *env* divergence prior to nAbs (median: 0.030% per month, range: 0.020–0.046% per month) and a large increase (median: 0.239% per month, range: 0.090–0.40% per month) concurrent with detection of nAbs (Fig 6, S1 Table). This translated to a fold increase in divergence ranging from 4.3–19. In one participant (CAP63), a substantial increase in the rate of *env* divergence was observed prior to detectable nAbs (0.20% per month) which decreased by the time nAbs were detected (0.12% per month). A single nAb escape mutation in CAP63 was responsible for the high rate of divergence prior to detectable nAbs (S1 Table, Fig 7). These results indicate diversification in early infection was largely linked with the emergence of nAb responses.

A more detailed analysis at each amino acid position identified a number of conserved amino acid changes prior to the detection of nAbs. To map these changes, we calculated the Jensen-Shannon divergence (D_JS_) over time which considers the dissimilarity between two probability distributions (i.e. the difference in entropy between two time-points) [27]. Examination of these participants revealed several sites with either raised D_JS_ prior to nAb responses (Fig 7, white arrows), or concurrent with them (Fig 7, blue arrows). These sites were evaluated to determine if they were associated with neutralization escape.

In three of the five participants (CAP63, 210 and 239), sites exhibiting raised D_JS_ were observed as early as between three and nine weeks prior to the detection of nAbs (Fig 2). The changes detected in CAP210 (A161V and V208I; both detected nine weeks prior to nAbs) and CAP63 (P397S) conferred resistance to nAbs (S3 Fig). We were not able to produce functional PSV with the S365L mutation observed in sequences from CAP239.

We also evaluated the mutations associated with raised D_JS_ detected after nAb responses. In CAP239 sequences, all changes observed (K106E, L278S, W459R, E463G, K463.03E) resulted in escape from the nAb response (Fig 1D). This was similar for the remaining two participants CAP45 and CAP88, where sites with raised D_JS_ (D462G in CAP45; K343E and K350E in CAP88) were associated with escape from the respective nAb response. Consequently, all the sites with raised D_JS_ that were explored were associated with rapid nAb escape, including sites that evolved prior to the detection of nAb responses (<1:45). In comparison, there was little evidence of selection pressure elsewhere in the region that could not be accounted for by nAb pressure.

## Discussion

ADCC responses may contribute to protection from HIV-1 infection, as suggested by the results of RV144 vaccine trial [2]. While this result was not recapitulated in the follow-up trial in South Africa (HVTN702), the outcomes of a number of preclinical studies suggest this antibody function plays an important role [28, 29]. Here, we examined the role of ADCC responses in natural infection to determine the effect of these antibodies on early viral evolution–a sensitive approach to measure the importance of a response. In one of five individuals, we identified virus escape from ADCC-mediated immune pressure in early infection. In this participant, ADCC responses and concomitant escape were detected in multiple escape pathways and persisted until 22 weeks post infection, suggesting ongoing immune pressure. However, we did not find escape associated exclusively with ADCC in the remaining four individuals investigated. In contrast, escape from nAbs in all five individuals was rapid, and often occurred prior to the development of detectable nAbs. Together these results suggest that early ADCC-mediating antibody responses exert immune pressure and can play a role in moulding viral populations. However, this early non-neutralizing antibody immune pressure is weaker than the subsequent nAb pressure.

This is the first study on non-neutralizing antibody escape in humans to utilize a replicating virus assay to identify ADCC responses. In this assay, the HIV Env trimer is presented on the surface of the cells, enabling the detection of responses to conformational epitopes. An earlier study, using linear peptides loaded on the surface of cells [20], provided evidence of escape from ADCC responses in chronic infection. In that study, Chung *et al*. identified escape from ADCC responses mapped to the C1 region, a common target of ADCC-mediating antibodies [30, 31]. Here, we identified one site in C1 (K97E) of CAP239 viruses which facilitated resistance to ADCC responses in early infection but had no effect on neutralization, suggesting this region was also targeted by nnAbs in our study.

We also identified ADCC escape at a single site in the C5 region (S481N) of CAP239 viruses. This is anterior to previously characterized ADCC-mediating nnAbs to the C5, which have typically been located close to the gp120 C terminus [32–34]. S481N appeared inaccessible to antibodies in the closed trimer model and did not have any effect on neutralization. The mutation was fixed in two of the three viral evolutionary pathways identified, suggesting that there was significant immune pressure on this site. However, while earlier viruses only escaped ADCC, later viruses also incorporated nAb escape mutations in V5, implying there was a dynamic adaptation to pressure from both responses. Two alternative escape pathways were observed after the detection of nAbs (from 19 wpi). The second pathway included viruses that escape both nAb and ADCC and persisted through to 37 wpi (the last time-point sequenced), suggesting ADCC-mediating antibody pressure, together with nAbs, continued to play a role in driving viral evolution up to this time-point.

However, the third pathway consisted of viruses that were resistant to nAbs but susceptible to ADCC responses. The persistence of these viruses, despite sensitivity to ADCC-mediating antibodies, suggests ADCC pressure on the viral Env was weak compared to nAb pressure. This may be due to the relative strength of nAb immune pressure or indicate dysfunction of effector cells (in particular, NK cells) in this individual, as has observed in acute HIV-1 infection [35, 36]. If the latter is the case, this would be less of a concern for future vaccines which elicit ADCC-mediating antibodies, as NK cell dysregulation is unlikely to occur in the context vaccination.

Richardson *et al*. identified high levels of ADCC activity in CAPRISA participants very early in infection [37]. Our study took advantage of this kinetics of antibody responses where we found that autologous ADCC-mediating antibody responses emerged in all five individuals prior to nAb responses. This allowed us to contrast selective pressure exerted by these two antibody functions. Using single genome and deep sequencing, we tracked the impact of early ADCC and nAb responses on viral *env* evolution. In three of the five participants, low levels of nAb escape were observed as early as two to nine weeks prior to the detection of nAbs, suggesting that even at very low titres nAb drive viral escape. This finding is consistent with a previous study by Bar *et al*., which found evidence of very low levels of escape variants (0.2–1.1%) in two individuals from the CHAVI acute infection cohort [38].

The temporal association of nAb development with viral escape may be due to the increased affinity and avidity of nAbs compared to ADCC-mediating antibodies. While we observed ADCC-mediating antibodies from enrolment, nAbs only became detectable later suggesting these responses require further B cell maturation to neutralize virions. Affinity maturation of nAb lineages has been observed in several longitudinal studies, where binding affinities of the initial antibody to the antigen are substantially lower than subsequent members of a lineage [39, 40]. This has also recently been shown for ADCC-mediating antibodies [41]. It is likely that ADCC-mediating antibodies continued to evolve after the detection of nAbs. However, deconvoluting the role of these ADCC-mediating antibodies on viral evolution after the development of nAbs is significantly more complicated. Further study of these responses using isolated monoclonal antibodies will be required to define the role of affinity maturation in this study.

Many nAbs can mediate ADCC, indicating that several Env epitopes are present on both free virions and on the surface of infected cells [42]. Here, a nAb escape mutation in the V5 (D462G) of viruses in one participant (CAP45) resulted in resistance to ADCC, suggesting these responses overlapped or were mediated by the same antibody. However, this pattern was not observed in CAP210 where two mutations had different effects on neutralization and ADCC: one mutation (A161V) resulted in resistance to nAbs but had no effect on ADCC, while the other mutation (V208I) resulted in resistance to ADCC responses but only partially escaped antibody neutralization. This suggests that neutralization and ADCC were mediated via different antibodies.

Numerous epitopes are present on the surface of infected cells as a result of the differences in Env conformations (open or closed trimers), or forms (e.g. gp41 stumps or monomeric gp120), compared to virions where neutralization is dependent on the Env trimer [43, 44]. In addition, the level of CD4 downregulation, which in this assay may be partially affected by impaired Nef activity [45, 46], is expected to impact the relative composition of Env conformations on the membrane of infected cells. However, we observed little difference in recognition of cells infected by the T/F or mutant viruses by control antibodies, CD4 downregulation and infectivity, suggesting these factors did not play a role in the differential recognition by plasma ADCC-mediating antibodies that we observed.

In conclusion, we demonstrate ADCC-mediating nnAb driven immune escape in early HIV-1 infection, showing that these responses can exert selective pressure on HIV-1, albeit only to a low extent. Here, viruses that were ADCC resistant were selected for in the absence of nAb pressure. However, after nAbs developed the virus evolved to preferentially escape nAb responses, often without escaping ADCC, indicating nAbs exert stronger selective pressure on the virus. While responses that control infection may be different from those that provide protection, the ability to force immune pressure provides an indication of the importance of an immune response. Our data adds to the cumulative evidence that while eliciting nAbs should be the primary goal of HIV-1 vaccine design, ADCC-mediating antibodies could contribute to increasing the protective efficacy of a vaccine.

## Methods

### Ethics statement

The CAPRISA 002 study received ethical approval from the Universities of KwaZulu-Natal (E013/04), Cape Town (025/2004), and the Witwatersrand (MM040202). All participants in this study provided written informed consent for study participation.

### CAPRISA 002 acute infection cohort participants

The CAPRISA 002 acute infection cohort, described previously [47, 48], was established in 2004 and recruited recently HIV-1 infected women, from Durban, KwaZulu-Natal, South Africa, prospectively. HIV-1-infected participants were recruited within 3 months of a previous HIV-1 negative test. Following detection of infection, plasma samples were taken weekly for 3 weeks, fortnightly until approximately 3 months post infection, monthly until approximately 1 year post infection, and quarterly thereafter. The time of infection was estimated as the mid-point between the last HIV-1 negative sample and the first HIV-1 positive sample, or 14 days prior to the first HIV-1 positive sample if the sample was RNA-positive, seronegative. Each participant was monitored for CD4 count and viral load.

### Cell lines

TZM-bl cells [24] were obtained from the NIH AIDS Research and Reference Reagent Program (NIH ARRRP, catalogue number 8129, contributed by John Kappes and Xiaoyun Wu). The HEK293T cell line was obtained from Dr George Shaw (University of Pennsylvania, Philadelphia, PA). All adherent cell lines were cultured at 37°C, 5% CO_2_ in DMEM containing 10% heat-inactivated Fetal Calf Serum (FCS) (Biochrom) with 50 μg/mL gentamicin (Lonza, Basel, Switzerland) and disrupted at confluency by treatment with 0.25% trypsin in 1 mM EDTA (Lonza). CEM.NKR_CCR5_ cells were obtained through the NIH AIDS Research and Reference Reagent Program (NIH ARRRP, catalogue number 4376, contributed by by Dr. Alexandra Trkola).

### Peripheral blood mononuclear cells

Peripheral blood mononuclear cells (PBMCs) from a single FcγRIIIa 158FV heterogenous individual were used as a source of effector cells. PBMCs were obtained from healthy HIV-1 seronegative donors and isolated by Ficoll gradient from buffy coats. Immediately after isolation, PBMCs were counted, resuspended in 10% DMSO, 20% FCS, 70% RPMI and cooled to -80°C at a rate of -1°C/h overnight. The next day, PBMCs were stored in liquid nitrogen storage.

### RT-PCR amplification and sequencing

HIV-1 RNA was purified from plasma using the Qiagen Viral RNA kit (Qiagen), and reverse transcribed to cDNA using Superscript III Reverse Transcriptase (Invitrogen, CA). PCR amplification of HIV-1 *env* genes was done using the single-genome amplification (SGA) approach previously described [49]. Briefly, single genome amplification (SGA) or end-point dilution was carried out on cDNA. Amplicons were directly sequenced using the ABI PRISM Big Dye Terminator Cycle Sequencing Ready Reaction kit (Applied Biosystems, Foster City, CA) and resolved on an ABI 3100 automated genetic analyzer. The full-length *env* sequences were assembled and edited using Sequencher v.4.0 software (Genecodes, Ann Arbor, MI). Multiple sequence alignments were performed using Clustal X (ver. 1.83) and manually edited using BioEdit (ver. 5.0.9).

### Cloning gp160 and mutagenesis

PCR amplification was carried out as above with the following exception: the second-round PCR reaction was repeated using the high-fidelity Platinum Taq DNA Polymerase (Invitrogen, Carlsbad, CA), together with 0.2 mM dNTPs (Invitrogen), 4 μM of Env 1A-Rx (5’- CACCGGCTTAGGCATCTCCTATAGCAGGAAGAA -3’) and EnvN (5’- CTGCCAATCAGGGAAAGTAGCCTTGT -3’) in a final volume of 20 μL. Amplicons were then cloned into the directional vector pcDNA3.1(+) (Invitrogen) per the manufacturer’s instructions. Site-directed mutagenesis was performed using the Stratagene QuickChange II kit (Stratagene). Env pseudoviruses were obtained by co-transfecting the *env* plasmid with pSG3ΔEnv [24] in HEK293T cells using PolyFect transfection reagent (Qiagen, Hilden, Germany). Pseudovirus-containing supernatants were harvested 48 h following transfection and clarified by 0.45 μm filtration and adjusted to 10% FCS (Biochrom). The 50% tissue culture infectious dose (TCID_50_) for each pseudovirus preparation was determined by infection of TZM-bl cells as previously described [47, 50].

### Construction of HIV-1 infectious molecular clones and virus preparation

HIV-1 infectious molecular clones (IMCs) encoding the *env* for early and mutant viruses in an isogenic proviral backbone were constructed as previously described [51], and also expressed the *Renilla* luciferase (LucR) reporter gene under the control of the HIV-1 Tat protein. Replication-competent reporter viruses like NL-LucR.T2A-C.CAP239TF.ecto and *env* mutants thereof are collectively referred herein as Env-IMC-LucR viruses. Originally designed to preserve all nine viral open reading frames by linking Nef expression to LucR via a ribosome-skipping T2A peptide, we later found Nef expression was reduced in primary CD4 T cells [45]. Replication-competent viruses were obtained by transfecting the Env-IMC-LucR plasmid in HEK293T cells using PolyFect transfection reagent (Qiagen). Virus-containing supernatants were harvested 48 h following transfection, clarified by 0.45 μm filtration and adjusted to 10% FCS (Biochrom). The 50% tissue culture infectious dose (TCID_50_) for each IMC preparation was determined by infection of TZM-bl cells as previously described [47, 50].

### Infection of CEM.NKR_CCR5_ cell line with HIV-1 Env-IMC-LucR

A total of 1 x 10^6^ cells were infected with 1 mL of virus inoculum by spinoculation at 2500 rpm for 2 h at 32°C in the presence of DEAE-Dextran (7.5 μg/mL). The cells were subsequently resuspended at 0.3 x 10^6^/mL and cultured for three days in complete medium containing 7.5 μg/mL DEAE-Dextran. On assay day, the infection was monitored by measuring the frequency of cells expressing intracellular p24. Assays performed using the Env-IMC-LucR-infected target cells were considered reliable if the percentage of viable p24+ target cells on assay day was ≥10%. Following 72 h of infection, cells were washed in PBS, dispensed in 96-well V-bottom plates at 1 x 10^5^ viable cells per well, and stained with a vital dye (LIVE/DEAD Fixable Aqua Dead Cell Stain, Invitrogen) to exclude non-viable cells from subsequent analyses. The cells were then washed twice with 250 μL per well of washing buffer (WB; PBS + 1% FBS) and incubated with Cytofix/Cytoperm (BD Bioscience) for 20 min at 4°C. The cells were then washed twice with 200 μL of 1% Cytoperm washing buffer. After the final wash, the anti-p24 Antibody (clone KC57-RD1; Beckman Coulter, Brea, CA) was added to a final dilution 1:400 and the plates were incubated for 30 min at 4°C. The plates were washed twice with WB, and the cells were resuspended in 200 μL 1% formaldehyde-PBS. The samples were acquired within 24 hours using the LSR II flow cytometer. A minimum of 10,000 total singlet events was acquired for each analysis. Gates were set to include singlet and live events. The appropriate compensation beads were used to compensate the spill over signal for the two fluorophores. Data analysis was performed using FlowJo 10.2 software (TreeStar). Uninfected CEM.NKR_CCR5_ and the chronically infected A1953 cell lines were used as negative and positive controls, respectively.

### Neutralization assay

Neutralization was measured, as previously described [47], by a reduction in luciferase gene expression after single round infection of TZM-bl cells with Env pseudo or infectious viruses. Plasma was heat inactivated prior to assays. Titers were calculated as the reciprocal plasma dilution which resulted in a 50% reduction (ID_50_) of the relative light units (RLU). Assays were repeated a minimum of three times.

### *Renilla* Luciferase-based ADCC assay

The LucR-based ADCC assay was conducted as described by Pollara *et al*. [52]. The day prior to the ADCC assay, PBMCs from a single FcγRIIIa 158FV donor (resulting in no effector cell variability) to be used as effectors in the assay were thawed in R10, counted and assessed for viability. PBMCs were resuspended in R10 supplemented with IL-15 (at a final concentration of 10 ng/mL) (Sigma) overnight to stimulate NK cells. On the day of the assay, infected CEM.NKR_CCR5_ cells were counted, assessed for viability (viability was ≥ 80% to be used in the assay) and the concentration was adjusted to 2 x 10^5^ viable cells/mL (5 x 10^3^ cells/well). Stimulated PBMCs were then counted, assessed for viability, pelleted and resuspended in the infected CEM.NKR_CCR5_ cells at a concentration of 6 x 10^6^ PBMCs/mL (1.5 x 10^5^ PBMCs/well) (effector: target cell ratio of 30:1). Heat-inactivated autologous plasma or controls were serially diluted. The effector/ target cell mix and antibody dilutions were plated in opaque 96-well half-area plates, centrifuged at 300 x g for 1 min after 30 min, and then incubated for 5.5 hrs at 37°C, 5.5% CO_2_ to allow ADCC-mediated cell lysis to proceed. After 5.5 hrs, ViviRen substrate (Promega) was diluted 1:500 in R10 and added 1:1 to the assay wells. The substrate generates luminescence only in live, infected cells; not in dead or lysed cells. The final readout was the luminescence intensity generated by the presence of residual intact target cells that have not been lysed by the effector population in the presence of ADCC-mediating antibodies. The percentage of specific killing was calculated using the formula

$$\%\;\mathrm{specific}\;\mathrm{killing}=\frac{\mathrm{RLU}\;\mathrm{of}\;\mathrm{target}+\mathrm{effector}\;\mathrm{well}‐\mathrm{RLU}\;\mathrm{of}\;\mathrm{test}\;\mathrm{well}}{\mathrm{RLU}\;\mathrm{of}\;\mathrm{target}+\mathrm{effector}\;\mathrm{well}}x\;100$$

In the analysis, the RLU of the target + effector wells represent lysis by NK cells in the absence of any source of antibody. Plasma from a seronegative donor was used as a negative control and purified IgG from individuals chronically infected with HIV-1 (HIVIG) or A300 (plasma from a chronically infected individual) were used as a positive control. Titers were calculated as the reciprocal of the highest plasma dilution or the lowest concentration of HIVIG which resulted in % specific killing above background signal. All experiments were repeated a minimum of three times.

### Infected cell antibody binding assay

Infected CEM.NKR_CCR5_ cells were obtained as described above. Cells incubated in the absence of virus (mock infected) were used as a negative infection control. Following infection, infected and mock infected cells were washed in PBS, dispensed into 96-well V-bottom plates at 2 x 10^5^ cells/well and incubated with 10 μg/mL HIVIG for 2 h at 37°C. Subsequently, cells were washed twice with 250 μL/well of wash buffer (1%FBS-PBS; WB) and stained with vital dye (Live/Dead Fixable Aqua Dead Cell Stain, Invitrogen) to exclude nonviable cells from subsequent analysis. Cells were then resuspended in 100 μL/well Cytofix/Cytoperm (BD Biosciences), incubated in the dark for 20 min at 4°C, washed in 1x Cytoperm wash solution (BD Biosciences) and co-incubated with anti-p24 antibody (clone KC57-RD1; Beckman Coulter) to a final dilution of 1:100 and a secondary FITC-conjugated antibody (goat anti-human IgG(H+L)-FITC, KPL) to a final dilution of 1:100, and incubated in the dark for 25 min at 4°C. Cells were washed three times with Cytoperm wash solution and resuspended in 125 μL PBS-1% paraformaldehyde. The samples were acquired within 24 h using a BD Fortessa cytometer. A minimum of 25,000 total events was acquired for each analysis. Gates were set to include singlet and live events. The appropriate compensation beads were used to compensate the spill over signal for the three fluorophores. Data analysis was performed using FlowJo 9.6.6 software (TreeStar). MFI from wells which included the secondary antibody alone (no mAb or plasma) were subtracted from samples to calculate the MFI specifically due to HIVIG binding. Plasma from a seronegative donor were used as negative controls. Experiments were repeated a minimum of three times.

### Structural modelling and glycosylation changes

Crystal structures of the HIV-1 Env trimer and gp41 (PDB IDs: 4NCO, 4TVP, and 2B4C) were used as templates to create models from the CAP239 gp160 sequences, which was carried out using Modeller [53] and the UCSF Chimera package [54]. Modelling was done in 10 replicates, and the best scoring model was chosen for further analysis. Basic mannose glycans were added at selected potential N-linked glycosylation sites using GlyProt [55].

### Deep sequencing library preparation and data processing

RNA extraction, cDNA synthesis and subsequent amplification were carried out using the primer ID method as described previously [26, 56], with the following modifications: cDNA synthesis primers were designed to bind to the C2 (HxB2: 6907–6883), C3 (HxB2: 7343–7318) or C5 (HxB2: 7655–7632) regions of the HIV-1 *env* respectively (depending on the region to be amplified). First-round amplification primers were designed to bind to the C1 (HxB2: 6654–6674), C2 (HxB2: 6950–6973) or C3 (HxB2: 7114–7135) regions respectively. This allowed amplification of the C1C2, C2C3 or C3C5 regions depending on the primer set used. Raw reads were processed using a custom pipeline housed within the University of Cape Town High Performance Computing core, as previously described [56].

## Supporting information

S1 Fig ADCC, binding and infection profiles of CAP239 T/F and mutant infected cells. **(A)** ADCC antibody titers against CAP239 TF, CAP239 TF K97E, or CAP239 TF S481N infected targets cells in the Luciferase ADCC assay using purified IgG from chronically infected individuals (HIVIG) as a source of antibody. **(B)** Binding of HIVIG to infected cells, measured by the median fluorescent intensity of the secondary antibody (goat anti-human IgG(H+L)-FITC). **(C)** Infectivity and CD4-downregulation of infected targets cells, as shown by flow plots (top) and bar graphs (bottom).
(TIF)

S2 Fig Relative frequencies of wildtype and mutant residues. The relative frequencies are shown as a percentage of consensus sequences generated. The WT is represented by a blue line and mutant residues by red lines. Confirmed escape mutations evaluated: 462Δ3 and D462G (in the V5) of the Env in CAP45; A161V (in V2) and V208I (in C2) of the Env in CAP210. The shaded region indicates the time from which the initial nAb response is first detected (IC_50_).
(TIF)

S3 Fig Neutralization kinetics and sites of escape in four CAPRISA 002 participants. Mutations were introduced into the T/F *env* pseudovirus, and neutralization assays were performed to determine the effect of the mutation on each response. The impact of each mutation on sensitivity to autologous nAb responses was tested using longitudinal plasma from each participant.
(TIF)

S1 Table Rate of divergence in the region targeted by the initial nAb response before and after the detection of nAbs. (DOCX)

S1 Data Spreadsheet containing all data used to generate figures. (XLSX)
