# Supplementary figures and images for: ADCC-mediating non-neutralizing antibodies can exert immune pressure in early HIV-1 infection
Source: PLoS Pathog. 2021 Nov 17;17(11):e1010046. doi: 10.1371/journal.ppat.1010046 (PMC8598021; doi:10.1371/journal.ppat.1010046)

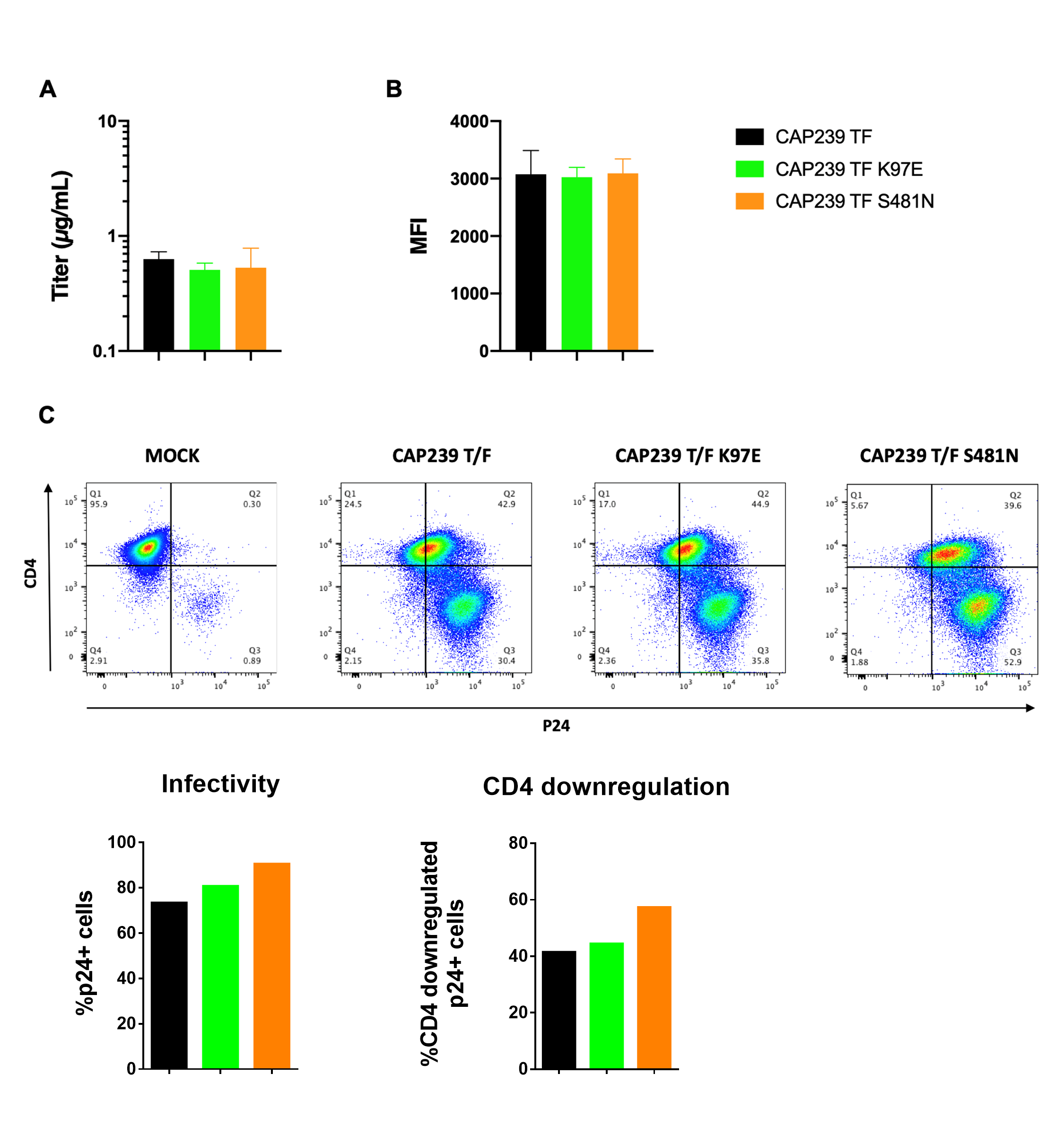

Supplement: S1 Fig — (A) ADCC antibody titers against CAP239 TF, CAP239 TF K97E, or CAP239 TF S481N infected targets cells in the Luciferase ADCC assay using purified IgG from chronically infected individuals (HIVIG) as a source of antibody. (B) Binding of HIVIG to infected cells, measured by the median fluorescent intensity of the secondary antibody (goat anti-human IgG(H+L)-FITC). (C) Infectivity and CD4-downregulation of infected targets cells, as shown by flow plots (top) and bar graphs (bottom). (TIF) [file ppat.1010046.s001.tif]

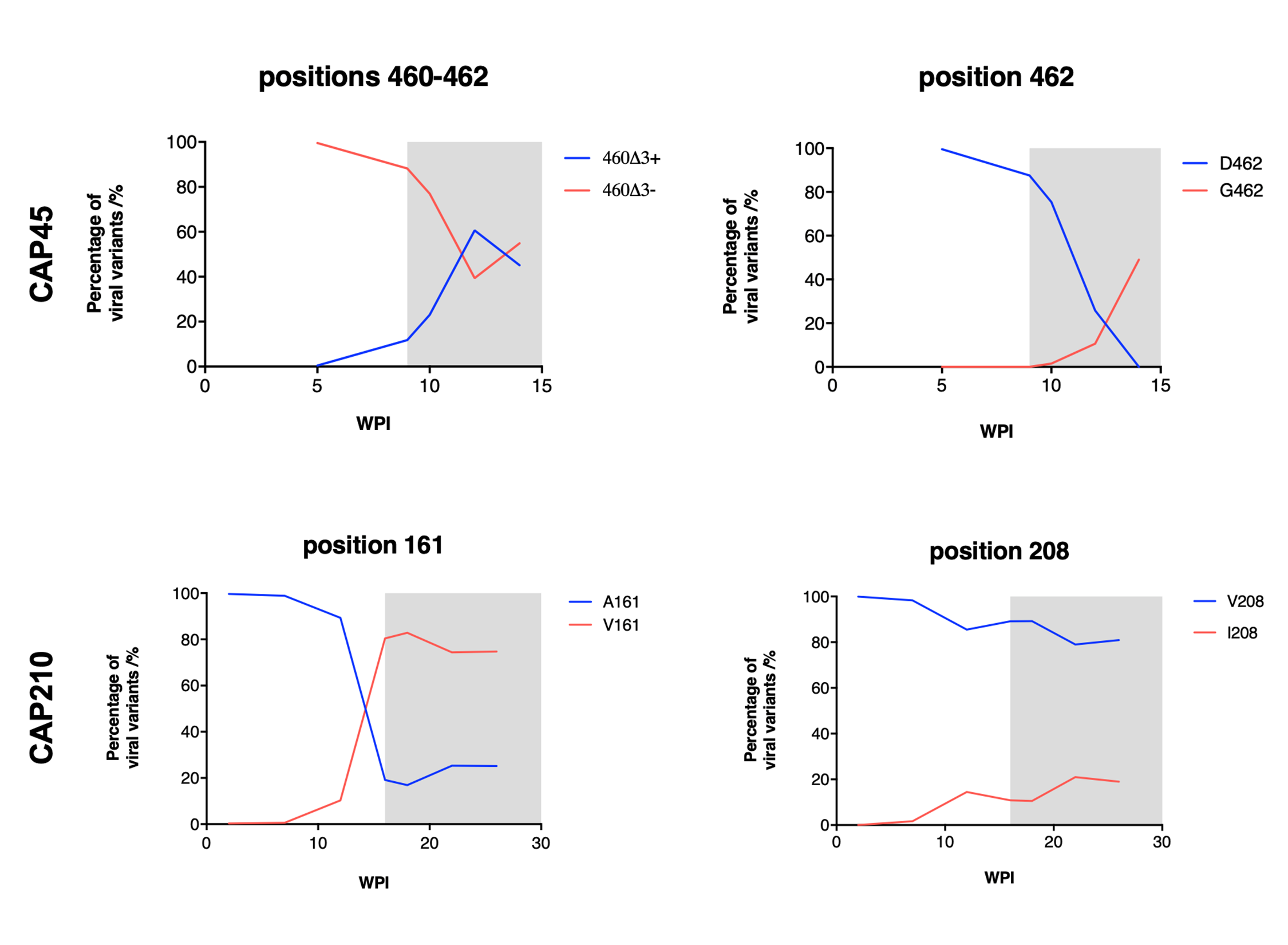

Supplement: S2 Fig — The relative frequencies are shown as a percentage of consensus sequences generated. The WT is represented by a blue line and mutant residues by red lines. Confirmed escape mutations evaluated: 462Δ3 and D462G (in the V5) of the Env in CAP45; A161V (in V2) and V208I (in C2) of the Env in CAP210. The shaded region indicates the time from which the initial nAb response is first detected (IC50). (TIF) [file ppat.1010046.s002.tif]

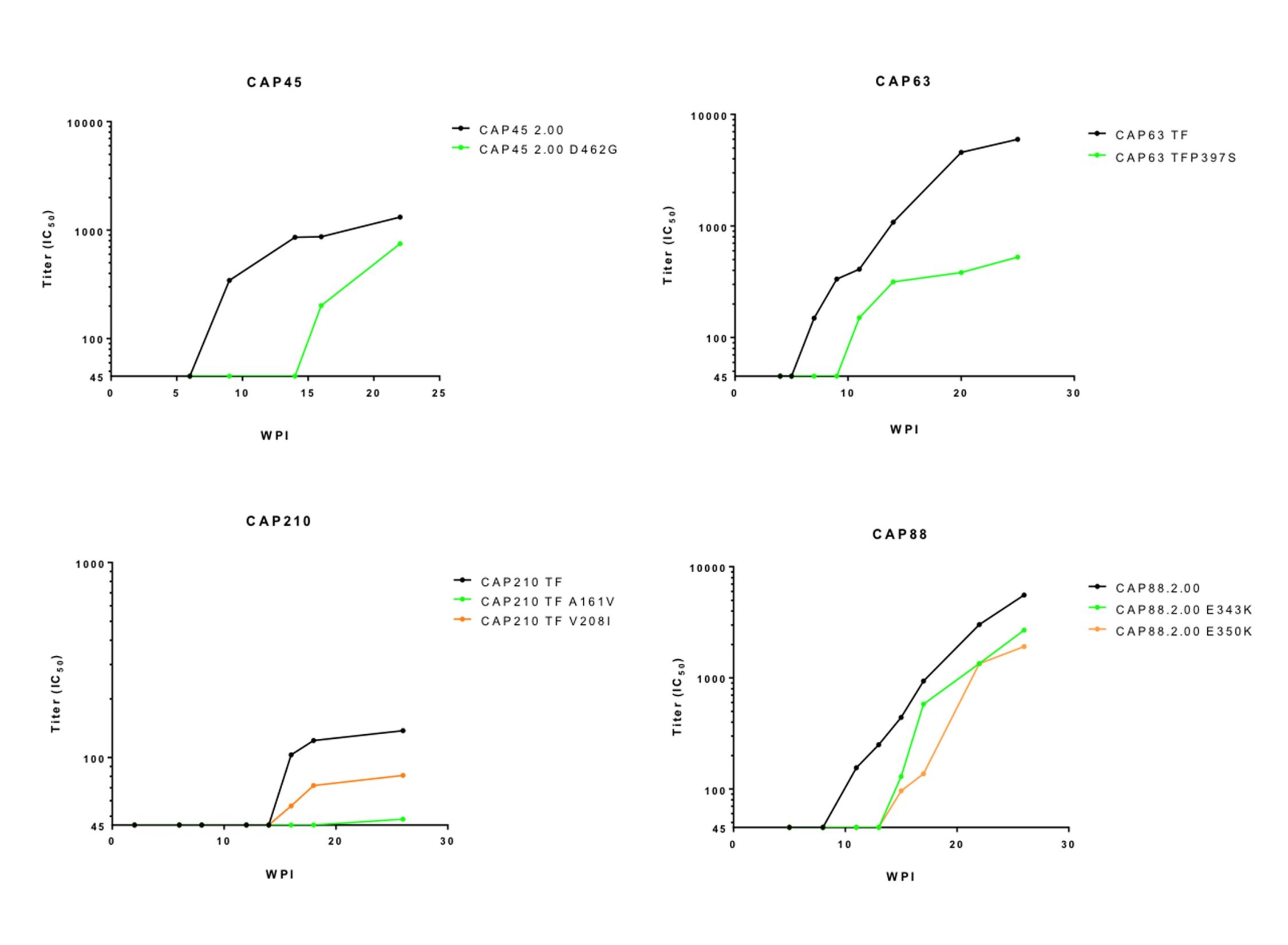

Supplement: S3 Fig — Mutations were introduced into the T/F env pseudovirus, and neutralization assays were performed to determine the effect of the mutation on each response. The impact of each mutation on sensitivity to autologous nAb responses was tested using longitudinal plasma from each participant. (TIF) [file ppat.1010046.s003.tif]
