# Supplementary material for: ADCC-mediating non-neutralizing antibodies can exert immune pressure in early HIV-1 infection
Source: PLoS Pathog. 2021 Nov 17;17(11):e1010046. doi: 10.1371/journal.ppat.1010046 (PMC8598021; doi:10.1371/journal.ppat.1010046)
Supplement: S1 Table — (DOCX) [file ppat.1010046.s004.docx]

**Table S1. Rate of divergence in the region targeted by the initial nAb response before and after the detection of nAbs.**

| **Participant** | **Env region** | **Last nAb negative time-point (wpi)** | **Average rate of divergence prior to detectable nAbs (per month)** | **Time-point of first detectable nAbs (wpi)** | **Average rate of divergence after detectable nAbs (per month)** | **Fold-increase in rate of divergence** |
| --- | --- | --- | --- | --- | --- | --- |
| **CAP45** | C3C5 | 5 | 0.020 | 9 | 0.090 | 6.0 |
| **CAP63** | C3C5 | 4 | 0.2 | 7 | 0.12 | 0.6 |
| **CAP88** | C2C3 | 8 | 0.0025 | 11 | 0.064 | 19 |
| **CAP210** | C1C2 | 12 | 0.040 | 16 | 0.17 | 4.3 |
| **CAP239** | C1C2 | 11 | 0.012 | 13 | 0.0097 | 0.8 |
|  | C2C3 |  | 0.046 |  | -0.038 | -0.04 |
|  | C3C5 |  | 0.037 |  | 0.307 | 8.3 |
